# Correction: Assembly principles of a unique cage formed by hexameric and decameric *E. coli* proteins

**DOI:** 10.7554/eLife.05179

**Published:** 2014-10-21

**Authors:** Hélène Malet, Kaiyin Liu, Majida El Bakkouri, Sze Wah Samuel Chan, Gregory Effantin, Maria Bacia, Walid A Houry, Irina Gutsche

Malet H, Liu K, El Bakkouri M, Chan SWS, Effantin G, Bacia M, Houry WA, Gutsche I. 2014. Fringe proteins modulate Assembly principles of a unique cage formed by hexameric and decameric *E. coli* proteins. *eLife* **3**:e03653. doi: 10.7554/eLife.03653. Published 5 August 2014

In the published article, the first sentence of the legend for Figure 2—figure supplement 1A stated that the RavA leg bound to the LdcI inner ring was shown in blue/dark blue and the RavA leg bound to the Ldcl outer ring in yellow/red.

The colour assignments should have been given the other way around: in the figure supplement, the RavA leg bound to the LdcI inner ring is shown in yellow/red and the RavA leg bound to the LdcI outer ring is in blue/dark blue.

The legend has been corrected accordingly.

